# The Incidence, Localization and Clinical Relevance of Arterial Fenestrations and Their Association to Brain Aneurysms: A Case–Control Study Based on the STROBE Guidelines

**DOI:** 10.3390/brainsci12101310

**Published:** 2022-09-28

**Authors:** Wojciech Czyżewski, Zofia Hoffman, Michał Szymoniuk, Patrycja Korulczyk, Kamil Torres, Grzegorz Staśkiewicz

**Affiliations:** 1 Department of Didactics and Medical Simulation, Medical University of Lublin, Chodźki 4, 20-093 Lublin, Poland; 2 Department of Neurosurgery and Pediatric Neurosurgery, Medical University of Lublin, Jaczewskiego 8, 20-090 Lublin, Poland; 3 Student Scientific Society, Medical University of Lublin, Al. Racławickie 1, 20-059 Lublin, Poland; 4 Student Scientific Association, Department of Neurosurgery and Pediatric Neurosurgery, Medical University of Lublin, Jaczewskiego 8, 20-090 Lublin, Poland; 5 I Department of Medical Radiology, Medical University of Lublin, Jaczewskiego 8, 20-090 Lublin, Poland

**Keywords:** fenestration, aneurysm, malformation

## Abstract

*Background:* Fenestrations are rare, but well-known, vascular variations of the cerebral arteries. They are mostly incidental, asymptomatic angiographic findings and might precipitate vascular lesions such as AVM, aneurysmal dilatation, or even ischemic symptoms. However, association between arterial fenestration and brain aneurysms has not been clearly established. *Objective:* To evaluate whether incidence of arterial fenestrations are associated with brain aneurysm development and investigate the prevalence and most-common localizations of arterial fenestrations of the human brain. *Design:* Case–control study. *Setting:* All patients examined by CT angiography in University Hospital No. 4 in Lublin from 2009 to 2019. *Patients:* Each patient showing at least one cerebral aneurysm was included in the case group and each patient without cerebral aneurysm on CT angiography was included in the control group. *Measurements:* CT angiography examinations were conducted using the standard protocol used in the 1st Department of Radiology, Medical University of Lublin, Poland. The database and statistical research were conducted by use of the Statistica software (ver. 13.3, Tibco Software Inc., Palo Alto, CA, USA). *Results:* A total of 6545 CTA examinations were included in the study. Most of the aneurysms were located on the MCA: 629 (38.59%), ICA: 466 (28.59%) and AComA: 192 (11.78%). Cerebral arterial fenestration showed a non-statistically significant elevated risk for brain aneurysms in the entire study population (OR: 1.157; 95% CI: 0.826–1.621; *p* = 0.39). Among 6545 cranial CTA examinations, cerebral vessel fenestration was found in 49 of them, which constituted 0.75%. The most common vascular fenestrations were those located in the ACA (30.61%), BA (30.61%) and AComA (22.45%), while other fenestrations occurred infrequently. There were no significant differences in the age of patients in the individuals with vascular fenestration (*p* > 0.05). VA fenestration was slightly more common in men (16.67%) than in women (5.41%). However, these differences were not statistically significant (*p* = 0.216). *Limitations:* Our study has several limitations, including selection bias regarding examined population. Second, we assume that the total number of fenestrations detected in our study was underestimated due to the limitations of the CT method in comparison to other radiologic modalities. *Conclusions:* Cerebral arterial fenestrations are rare vascular malformations. The ACA is the most common localization of fenestrations, followed by BA and AComA. Fenestrations of cerebral arteries insignificantly increase the risk of cerebral aneurysm formation. Further prospective studies are necessary to make this association more precise.

## 1. Introduction

The cerebral vascular system was firstly illustrated by English scientist and architect Christopher Wren in the notable piece of work by Thomas Willis, “*Cerebri Anatome*”, published in 1664 [1]. Despite the fact that first cases of an incomplete circle were described relatively early, the first references to its variations did not occur until the twentieth century [2]. 

The circle of Willis has been investigated by numerous scientists ever since. The classic form consists of two ICAs and their anastomotic connections with the vertebrobasilar system. It is vital for any neurosurgeon to become familiar with the circle’s variations, as the classic-type occurs only in 5–30% of population [3]. Furthermore, any cerebral arterial variations such as fenestrations, duplications or persistent fetal arteries may play important roles in various cerebrovascular diseases; in consequence, they ought to be observed during brain surgeries [4,5].

When two distinct vessels with different origins do not share one distal convergence, the anomaly is then called arterial duplication [6], while vascular fenestrations are partial duplications within a vessel segment, which results in two distinct endothelium-lined channels that rejoin distally [7,8]. 

Fenestrations are rare, but well-known vascular variations of the cerebral arteries that most frequently occur in the anterior communicating artery, followed by the vertebrobasilar system, the anterior cerebral artery, the middle cerebral artery and the posterior cerebral artery [9]. Less frequently, fenestrations are found in the basilar artery or vertebral artery [10,11]. Although they are mostly incidental, asymptomatic angiographic findings, with a reported incidence of 0.03 to 1% [12], they might precipitate vascular lesions such as AVM, aneurysmal dilatation or even ischemic symptoms. Their association with aneurysm formation is due to turbulent flow caused by defects of tunica media at both ends of the fenestrated segments [13,14]. Increased hemodynamic stress precipitated by the anomaly, along with the absent media, are the main reasons for the higher occurrence of aneurysms in patients with vascular fenestrations [15]. 

The aim of our study is to evaluate an association between arterial fenestration prevalence and brain aneurysm development, based on CT angiography scans. Moreover, we assessed the frequency of prevalence and determined the most frequent locations of cerebral vascular fenestrations.

## 2. Materials and Methods

### 2.1. Study Design

This retrospective case–control study has been conducted according to Strengthening the Reporting of Observational Studies in Epidemiology (STROBE) guidelines [16]. For details, see Supplementary File S1.

### 2.2. Study Population and Setting

To investigate the prevalence and localizations of arterial fenestrations of the human brain and their association with brain aneurysms, we retrospectively evaluated cranial CT angiography (CTA) examinations registered at University Hospital No. 4 in Lublin, Poland, in the years 2009–2019.

The study size was determined by the number of cases examined during the study period. A total of 7251 CTA examinations performed with various indications were evaluated. The exclusion criterion of the study was improper visualization of vessels on CTA images, and 706 records were excluded from the study for that reason. Therefore, 6545 CTA examinations were included in the study, of which 1166 (17.8%) cases showing at least one cerebral aneurysm were included in the case group and 5379 (82.2%) cases without cerebral aneurysms on CTA were included in the control group.

The prevalence and most common localizations of arterial fenestrations were investigated among all patients included in the study.

### 2.3. Imaging Data

CT angiography examinations were conducted using standard protocol used in the 1st Department of Radiology, Medical University of Lublin, using 64-row and 256-row scanners (General Electric Medical Systems): scan range from the C1 to the vertex using tube voltage 120 kVp, and automatic tube-current modulation. The SmartPrep tracking technique was used with 50 mL of iodinated contrast agent followed by 40 mL saline bolus, injected at 5 mL/s. Contiguous sections were reconstructed with 0.5 mm slice thickness.

Images were evaluated on a dedicated workstation (Advantage Workstation 4.3, GEMS) by radiologists with at least 6 years of experience in CTA studies. Analysis included assessment of axial scans, 3 mm and reformations: maximum intensity projections (MIP) and volume rendering (VR).

### 2.4. Statistical Analysis

The database and statistical research were conducted by use of the Statistica software (ver. 13.3, Tibco Software Inc., Palo Alto, CA, USA). The normality of the distribution of quantitative variables was tested using the Shapiro–Wilk test. The value of the analyzed measurable variables was presented using the mean, standard deviation, median, lower and upper quartiles, as well as the minimum and maximum. Non-measurable parameters are presented numerically and as a percentage. The Kruskal–Wallis test was used to compare the age of the groups. In the case of qualitative variables, comparisons between individual features were made using the test of the existence of differences between two structure indices, while the chi-square test was used to test the relationship between these features. Univariate logistic regression analysis was performed to estimate odds ratio (OR) and 95% confidence interval (95% CI) to evaluate association between arterial fenestrations and brain aneurysms. In all cases, the level of significance was set at *p* < 0.05.

## 3. Results

### 3.1. Case Group (1 Tabela)

The case group included 778 women (66.7%) and 388 men (33.3%). The mean age of women in this group was 57.48 ± 13.71, and for men 56.32 ± 14.335. The number of patients in whom multiple aneurysms were detected in the head CTA study was 326. Among them, 231 were female and the remaining 95 were male. The majority of patients with multiple aneurysms had two aneurysms (226 people), while three aneurysms occurred in 70 people and four in 21 patients. Five or six aneurysms occurred in a total of nine patients. The group of patients with multiple aneurysms accounted for 27.96 % of all 1166 patients diagnosed with this type of vascular anomaly. The highest number of aneurysms were located in the MCA, ICA and AComA with 594, 428 and 186, respectively. Detailed characteristics of patients from the case group are presented in Table 1.

### 3.2. Association between Cerebral Arterial Fenestrations and Brain Aneurysms

Among 1166 patients from the case group, 11 (0.94%) presented fenestrations and 1155 (99.06%) presented no such finding. In the case of 38 (0.71%) patients from the control group, arterial fenestrations were found while 5341 (99.29%) controls did not demonstrate the presence of such. Cerebral arterial fenestration showed a non-statistically significant elevated risk for brain aneurysms in the entire study population (OR: 1.157; 95% CI: 0.826–1.621; *p* = 0.39) (Table 2).

We also investigated the relation between location of fenestration and occurrence of a particular aneurysm, and found no correlation. In more than 50% of the cases of coexistence, anterior cerebral artery aneurysm was involved with a fenestration located on various vessels (Table 3).

### 3.3. Prevalence and Localization of Cerebral Fenestrations

Among 6545 cranial CTA examinations, cerebral vessel fenestration was found in 49 of them, which constituted 0.75%. One of the patients was diagnosed with two fenestrations, both on the same vessel (LICA). There were 29 (75%) women and 12 (25%) men in the described cases.

**Figure 1:**
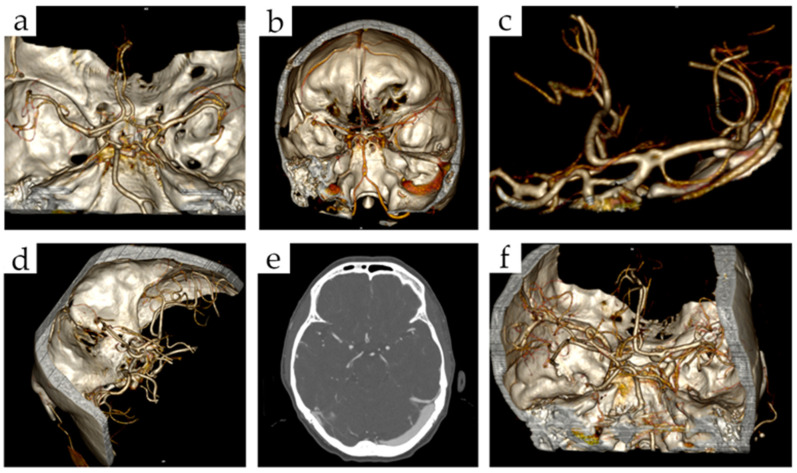
Cerebral arterial fenestrations on CT-3D reconstructions: (**a**) right ACA fenestration, (**b**) BA fenestration, (**c**) left MCA fenestration, (**d**) AComA fenestration, (**e**) AComA fenestration on CT angiography, (**f**) right MCA fenestration. (Abbreviations: ACA—anterior cerebral artery, BA—basilar artery, MCA—middle cerebral artery, AComA—anterior communicating artery).

**Figure 2:**
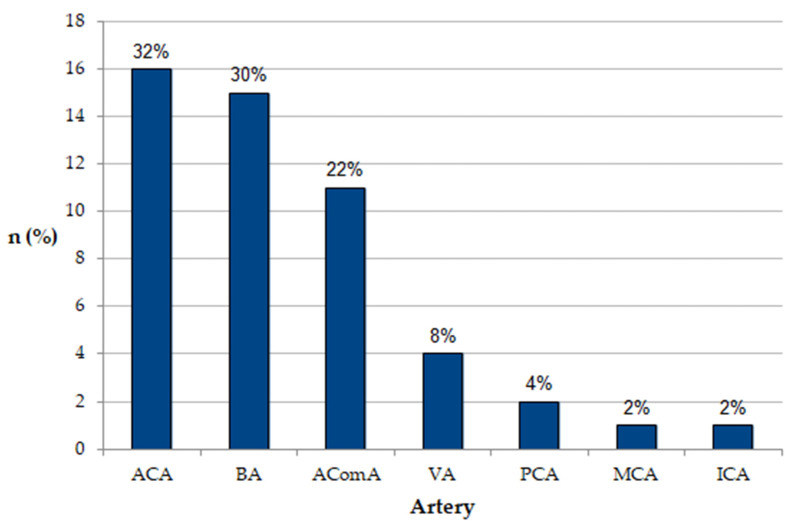
Incidence of fenestration by arteries. In one case, patient possessed two fenestrations, both on ACA. Abbreviations: ACA—anterior cerebral artery, BA—basilar artery, AComA—anterior communicating artery, VA—vertebral artery, PCA—posterior cerebral artery, MCA—middle cerebral artery, ICA—internal carotid artery.

The study showed that the most common vascular fenestrations were those located in the ACA (30.61%) (Figure 1a), BA (30.61%) (Figure 1b) and AComA (22.45%) (Figure 1d,e), while other fenestrations occurred infrequently (Figure 1c,f and Figure 2). There was only 1 patient of all the 6645 examined by CTA who had more than one fenestration, which were two ACA fenestrations.

### 3.4. Age and Gender

The patients with ICA fenestration had the lowest age, and the patients with MCA had the highest. Median patient age for the most common fenestrations was as follows: ACA was 60 years, BA was 46 years and AComA was 63 years. Statistical analysis did not reveal significant differences in the age of patients in the groups with individual vascular fenestration (*p* > 0.05) (Table 4).

It was shown that the occurrence of different types of fenestration was similar between women and men, except for VA, as this type was slightly more common in men (16.67%) than in women (5.41%). However, these differences were not statistically significant (*p* = 0.216) (Table 5).

## 4. Discussion

The results regarding the occurrence of cerebral vascular fenestration vary considerably among different studies. Moreover, it seems that results are largely influenced by the type of study conducted. In this study, fenestrations were found in 49 patients among 6545 CTA scans performed, which constitutes 0.75%; that is much less than reported in other studies based on CT (3.5–12.9%), MR (2.8–3.0%) and angiographic (22.9–28%) imaging, and significantly less than that indicated by post-mortem studies [17,18,19,20,21,22]. The above percentage discrepancies are due to the different sensitivities of each of these methods. Small vascular malformations may be difficult to notice on CT as well as MRI, but can be effectively visualized in DSA utilizing three-dimensional reconstruction [23]. The median age of examined patients for the most common fenestrations was 55 years for ACA, 48 years for BA and 57 years for AComA, which confirms the results obtained in other scientific studies, indicating a more frequent presence of vascular fenestration in the elderly [24]. However, taking into consideration the fact that imaging-examination studies of the head are more often performed on older people than on younger population, it can be concluded that the detectability of vascular fenestration is greater in the elderly than their presence.

The study shows that of all 6545 patients who underwent CTA of the head in years 2009–2019, 1166 suffered from aneurysm. This constitutes 17.8% of the examined patients, which is far more than was estimated in the general population: 2–6% [25,26]. In retrospective angiographic studies, the incidence of aneurysms was approximately 3.7%, and in prospective angiographic studies, 6%. [27].

It should also be added that a significant proportion of vascular malformations are found accidentally or at the time of a problem with another related vascular anomaly, e.g., in the event of an aneurysm rupture. This kind of discrepancy in the results may be caused by the fact that the CTA scans of patients described in this study and considered in calculating the statistical data were conducted due to the occurrence of indications for this type of imaging—i.e., neurologic symptoms of a suspected vascular origin—which created selection bias. Nonetheless, studies excluding this issue do not exist, as examinations that aim to evaluate cerebral vessels are rarely done on healthy individuals. A large prospective study that aims to assess the general population and that utilizes non-invasive angio-MRI examination is necessary to properly address the hypothetical correlation of the above-mentioned vascular malformations.

The modern literature delivers numerous examples of studies that aimed to evaluate coexistence of arterial fenestrations with other vascular malformations, along with their reciprocal relationship. Nevertheless, no universal consensus has been established in that matter so far.

Van Rooij et al. examined 140 patients after spontaneous subarachnoid hemorrhage using DSA and found 210 aneurysms. In this group, 45 fenestrations were detected in 33 individuals which comprised a vast 24% of the studied population. A total of 25% of patients with AComA aneurysm had coexistent fenestration on the same vessel [24]. Bożek et al. analyzed retrospectively 1140 CTAs and did not find correlation between both malformations [23]. In a meta-analysis conducted by Cooke et al., fenestration was found in 2.1% of angiograms performed. From these, 60.5% of patients had an aneurysm, 19.6% on the same artery [17]. The occurrence of SAH was slightly higher in patients with fenestration immediately associated with the aneurysm (66.7% vs 58.6%) although without statistical significance (*p* = 0.58). Zhen-Kui et al. analyzed 4652 angio-MRIs of patients suspected of vascular disease. The prevalence of both fenestration and an aneurysm reached 17% in comparison to aneurysms without fenestration, with statistical significance (*p* = 0.0064) [18]. The same observations were noted by Gao et al. using CT to evaluate arteries of posterior circulation [28]. The largest meta-analysis to date was presented by Guo et al. in 2018 [7]. According to their research, fenestration increased the risk of developing an aneurysm by 2.43 times in a group of patients examined for various reasons excluding SAH. However, in the SAH group this association was not observed [7]. Interesting observations were delivered by Krystkiewicz et al. in a study performed on 333 formalin-fixed brains. There, fenestrations were found in 41% of specimens, and aneurysms in 8%. An aneurysm correlated with a fenestration was detected in 2% (*p* = 0.18) of all specimens, leading the authors to proclaim the coexistence between these malformations to be incidental in character [29].

In our study, similarly to the cited meta-analysis, detection of fenestration increased the risk of developing an aneurysm; however, due to the limited number of cases, these results are not statistically significant (OR: 1.157; 95% CI: 0.826–1.621; *p* = 0.39). Nonetheless, identification of fenestration, in our opinion, should alert both the examined individuals as well as clinicians. According to some sources, however, fenestration itself not only potentially heralds other vascular malformation, but due to the alteration of local blood flow patterns, flow rate or pressure etc. can lead to cerebral ischemia in unfavorable circumstances.

Detection of fenestration as a single cause of ischemic syndrome is rare, particularly since most of evidence available in the literature is based on low-number case studies. Okudera et al. hypothesized a relationship between fenestration of the sphenoidal portion of the middle cerebral artery with cerebral infarction in a territory of lenticulostriate arteries [30], whereas Nasel et al. suspected fenestration of the cervical segment of the internal carotid artery to be responsible for transient ischemic attacks [31]. Meinel et al., Kloska et al., Gold et al. and Palazzo et al. suggest a similar pathologic process regarding posterior circulation [32,33,34,35]. Otmani reported a similar experience in nine cases of basilar-artery-fenestration-related brainstem infarctions [36]. Other authors had similar conclusions [37]. Interestingly, Ye et al. found a much higher incidence of ischemic events in patients with fenestrations of vertebrobasilar origin in comparison with those of anterior circulation (*p* < 0.05) [38].

The exact mechanism of fenestration-related ischemia is yet to be discovered, with many authors trying to assess the pathology as well as potential risk factors [39]. Miyamoto et al. indicate toward the role of dissection and local thrombus formation at the site of malformation [40].

Our study has several limitations, including selection bias regarding the examined population. Further prospective studies that aim to evaluate healthy as well as vascular-disease-suspected patients are needed to properly estimate the prevalence of vascular fenestrations as well as aneurysms. Second, we assume that the total number of fenestrations detected in our study was underestimated due to the limitations of the CT method in comparison to other radiologic modalities.

Taking all studies into consideration, we strongly believe that the overall prevalence of arterial fenestrations is much higher than reported in CT-, MRI- and DSA-based studies, as Krystkiewicz et al. proved in their specimen study. Adding the data available from meta-analyses [7], a correlation between aneurysms and fenestrations ought not be omitted from clinical analysis in cases of neurological diagnosis, prophylaxis and treatment, nor from the operating field, including endovascular malformation treatment and surgery.

## 5. Conclusions

Cerebral arterial fenestrations are rare vascular malformations. The ACA is the most common localization of fenestrations, followed by BA and AComA. Fenestrations of cerebral arteries insignificantly increase the risk of cerebral aneurysm formation, although further prospective studies are necessary to make this association more precise [9].

## Figures and Tables

**Table 1:** Case group characteristics.

	Male	Female	Total Cases/Aneurysms
**Mean age**	56.32 ± 14.34	57.48 ± 13.71	N/A
**Number of cases**	388	778	1166
Single aneurysms	293	547	840
Multiple aneurysms	95	231	326
Two aneurysms	75	151	226
Three aneurysms	12	58	70
Four aneurysms	7	14	21
Five aneurysms	1	7	8
Six aneurysms	0	1	1
**Localization of aneurysms**			
MCA	170	424	594
ICA	110	318	428
AComA	83	103	186
BA	43	71	114
ACA	42	60	102
VA	5	28	33
Pericallosal	12	17	29
PCA	14	12	26
PComA	3	9	12
PICA	2	1	3
Ophthalmic	0	2	2
Meningeal	0	1	1
Frontobasal	0	1	1
Anterior Choroidal	0	1	1
Callosomarginal	1	0	1
Total aneurysms	485	1048	1533

Abbreviations: MCA—middle cerebral artery, ICA—internal carotid artery, AComA—anterior communicating artery, BA—basilar artery, ACA—anterior cerebral artery, VA—vertebral artery, PCA—posterior cerebral artery, PComA—posterior communicating artery, PICA—posterior inferior cerebellar artery.

**Table 2:** Association between cerebral arterial fenestrations and brain aneurysms (contingency 2 × 2 table).

	Cases	Controls	Total
**Fenestration**	11 (0.94%)	38 (0.71%)	49
**No fenestration**	1155 (99.06%)	5341 (99.29%)	6496
**Total**	1166	5379	6545
Statistical analysis: **OR**: 1.157; **95%CI**: 0.826–1.621; ***p*** = 0.39			

**Table 3:** Occurrence of aneurysms in patients with various types of vascular fenestration.

Aneurysm, *n* (%)	Fenestration, *n* (%)					Total
	Right ACA	Right VA	BA	Right MCA	AComA	
**Right ICA**	0 (0.00)	1 (9.09)	0 (0.00)	0 (0.00)	0 (0.00)	1 (9.09)
**Right MCA**	2 (18.18)	0 (0.00)	0 (0.00)	1 (9.09)	1 (9.09)	4 (35.36)
**AComA**	1 (9.09)	0 (0.00)	0 (0.00)	0 (0.00)	0 (0.00)	1 (9.09)
**Left ICA**	1 (9.09)	0 (0.00)	1 (9.09)	0 (0.00)	0 (0.00)	2 (18.18)
**BA**	0 (0.00)	0 (0.00)	1 (9.09)	0 (0.00)	0 (0.00)	1 (9.09)
**Right PICA**	1 (9.09)	0 (0.00)	0 (0.00)	0 (0.00)	0 (0.00)	1 (9.09)
**Right PComA**	1 (9.09)	0 (0.00)	0 (0.00)	0 (0.00)	0 (0.00)	1 (9.09)
**Total**	6 (54.55)	1(9.09)	2 (18.18)	1 (9.09)	1 (9.09)	11 (100.00)
Statistical analysis: **Chi-square** = 17.390; ***p*** = 0.832						

Abbreviations: ACA—anterior cerebral artery, BA—basilar artery, AComA—anterior communicating artery, VA—vertebral artery, PCA—posterior cerebral artery, MCA—middle cerebral artery, ICA—internal carotid artery, PICA—posterior inferior cerebellar artery, PComA—posterior communicating artery.

**Table 4:** Patient age by type of cerebral arterial fenestrations.

Artery	Number of Fenestrations	Mean Age	Median Age
ACA	15	54.6 ± 16.37	60
BA	15	47.73 ± 19.48	46
AComA	11	56.64 ± 22.21	63
VA	4	41.5 ± 16.54	45
PCA	2	46 ± 2.83	46
MCA	1	70	70
ICA	1	17	17
Statistical analysis: **H** = 7.552; ***p*** = 0.273			

Abbreviations: ACA—anterior cerebral artery, BA—basilar artery, AComA—anterior communicating artery, VA—vertebral artery, PCA—posterior cerebral artery, MCA—middle cerebral artery, ICA—internal carotid artery.

**Table 5:** Gender of patients by type of vascular fenestration.

Artery	Females		Males		*p* Value
	*n*	%	*n*	%	
ACA	12	32.43	3	25	**0.626**
BA	12	32.43	3	25	**0.628**
AComA	8	21.62	3	25	**0.807**
VA	2	5.41	2	16.67	**0.216**
PCA	1	2.7	1	8.33	**0.392**
MCA	1	2.7	0	0	N/A
ICA	1	2.7	0	0	N/A

Abbreviations: ACA—anterior cerebral artery, BA—basilar artery, AComA—anterior communicating artery, VA—vertebral artery, PCA—posterior cerebral artery, MCA—middle cerebral artery, ICA—internal carotid artery, N/A—non-applicable.

## Data Availability

The data presented in this study are available on request from the corresponding author. The data are not publicly available due to local regulations.

## Supplementary Materials

The following supporting information can be downloaded at: https://www.mdpi.com/article/10.3390/brainsci12101310/s1, Supplementary File S1: “STROBE Statement—Checklist of items that should be included in reports of case-control studies”.
